# Superior cycle performance and high reversible capacity of SnO_2_/graphene composite as an anode material for lithium-ion batteries

**DOI:** 10.1038/srep09055

**Published:** 2015-03-12

**Authors:** Lilai Liu, Maozhong An, Peixia Yang, Jinqiu Zhang

**Affiliations:** 1 State Key Laboratory of Urban Water Resource and Environment, School of Chemical Engineering and Technology, Harbin Institute of Technology, Harbin 150001, China; 2 College of Environmental and Chemical Engineering, Heilongjiang University of Science and Technology, Harbin 150022, China

## Abstract

SnO_2_/graphene composite with superior cycle performance and high reversible capacity was prepared by a one-step microwave-hydrothermal method using a microwave reaction system. The SnO_2_/graphene composite was characterized by X-ray diffraction, thermogravimetric analysis, Fourier-transform infrared spectroscopy, Raman spectroscopy, scanning electron microscope, X-ray photoelectron spectroscopy, transmission electron microscopy and high resolution transmission electron microscopy. The size of SnO_2_ grains deposited on graphene sheets is less than 3.5 nm. The SnO_2_/graphene composite exhibits high capacity and excellent electrochemical performance in lithium-ion batteries. The first discharge and charge capacities at a current density of 100 mA g^−1^ are 2213 and 1402 mA h g^−1^ with coulomb efficiencies of 63.35%. The discharge specific capacities remains 1359, 1228, 1090 and 1005 mA h g^−1^ after 100 cycles at current densities of 100, 300, 500 and 700 mA g^−1^, respectively. Even at a high current density of 1000 mA g^−1^, the first discharge and charge capacities are 1502 and 876 mA h g^−1^, and the discharge specific capacities remains 1057 and 677 mA h g^−1 ^after 420 and 1000 cycles, respectively. The SnO_2_/graphene composite demonstrates a stable cycle performance and high reversible capacity for lithium storage.

Lithium-ion batteries (LIBs), as power sources for portable electronic devices, mobile communication devices and electric/hybrid vehicles, have attracted tremendous attention due to their high energy density, high working voltage and excellent cycle life. Graphite is the most widely used commercial anode material. However, with a theoretical specific capacity of 372 mA h g^−1^, graphite cannot meet the increasing demand for high capacity batteries. Therefore, the development of new alternative anode materials with higher performance is desired. Graphene, a one-atom-thick planar sheet of sp^2^-bonded carbon with a theoretical lithium storage capacity of 744 mA h g^−1^, has been widely studied for potential application in LIBs because of its unique properties, such as superior electronic conductivity, high theoretical specific surface area exceeding 2600 m^2^ g^−1^, and excellent mechanical properties^1^,^2^. Recently, chemically modified graphene with high surface area has been an attractive choice for synthesizing hybrid nanomaterials with the aim of improving their capacities, examples including CuO/graphene, Cu_2_O/graphene, CoO/graphenen, Co_3_O_4_/graphene, Fe_2_O_3_/graphene, Fe_3_O_4_/graphene, Mn_3_O_4_/graphene, NiO/graphene, SnO_2_/graphene, TiO_2_/graphene, VO_2_/graphene, and V_3_O_7_ nanowire templated graphene scrolls^3^,^4^,^5^,^6^,^7^,^8^,^9^,^10^,^11^,^12^,^13^,^14^,^15^,^16^. When used as anode materials for LIBs, these composites exhibited excellent electrochemical performances. Graphene sheets with various nanoparticles were used as anode materials for LIBs, which not only enhanced the unique properties of graphene and nanoparticles but also added novel functionality and properties due to the interaction between the materials. Graphene sheets could buffer the volume changes of nanoparticles and prevent them from conglomerating. Conversely, the nanoparticles can avert stacking of graphene sheets. SnO_2_ is considered as one of the most promising anode material substitutes due to its high theoretical specific capacity (782 mA h g^−1^) and low potential for lithium alloying^17^,^18^,^19^,^20^,^21^. Therefore, much research of SnO_2_/graphene composites used as an anode material has been conducted. Various approaches to fabrication of SnO_2_/graphene composites were reported, such as gas-liquid interfacial reaction, co-precipitation, in situ chemical synthesis, in situ oxidation route, hydrothermal, laser irradiation, microwave and ultrasonication methods^22^,^23^,^24^,^25^. Lian *et al.*^14^ prepared a SnO_2_/graphene composite by a gas-liquid interface reaction that exhibited a high reversible specific capacity when used as an anode material for LIBs. Zhao *et al.*^22^ introduced a graphene/SnO_2_ composite by the bivalent tin ion-assisted reduction method. Electrochemical measurement results showed that the composite could deliver a reversible capacity of 775.3 mA h g^−1^ and capacity retention of 98% after 50 cycles. Zhu *et al.*^23^ reported that a reduced graphene/tin oxide composite was synthesized by homogeneous co-precipitation and had capacities of 2140 and 1080 mA h g^−1^ at 100 mA g^−1^ for the first discharge and charge, respectively. Moreover, the composite exhibited good capacity retention with 649 mA h g^−1^ after 30 cycles. Yao *et al*.^16^ synthesized by an in situ chemical synthesis approach a SnO_2_/graphene composite with a reversible capacity of 765 mA h g^−1^ for the first cycle. Recently, several varieties of graphene/SnO_2 _nanoparticle composites have been reported as anode materials for LIBs, such as flower-like SnO_2_, SnO_2_ nanorods, SnO_2_ hollow nanosphere composites, ternary hybrids of graphene/SnO_2_/Au, SnO_2_-graphene-carbon nanotube mixtures and graphene/carbon nanosphere composites^24^,^25^,^26^,^27^,^28^,^29^,^30^. These SnO_2_/graphene hybrids used as anode for LIBs had different structures and exhibited excellent electrochemical performance. However, the cycling stability performance for LIBs at high current densities need to be improved. In addition, most of the preparation methods of these materials used dried neutral graphene oxide as a precursor material and required auxiliary reagents, which result in complex and time-consuming processes. Therefore, an easy and efficient method for synthesizing graphene/metal oxide composites with high capacity for LIBs should be developed.

In this study, we report a one-step microwave-hydrothermal method for the synthesis of SnO_2_/graphene composite with a microwave reaction system. This method features are quick heating, easily controlled pressure and temperature, high yield rate, and good homogeneity. In reaction process, a precursor material of liquid graphene oxide is reduced in situ, and a bivalent tin ion is oxidized without auxiliary reagents. The uniform composite shows high specific capacity and excellent cycling stability performance for LIBs at high current densities.

## Results

### Microstructural characterization

**Figure 1:**
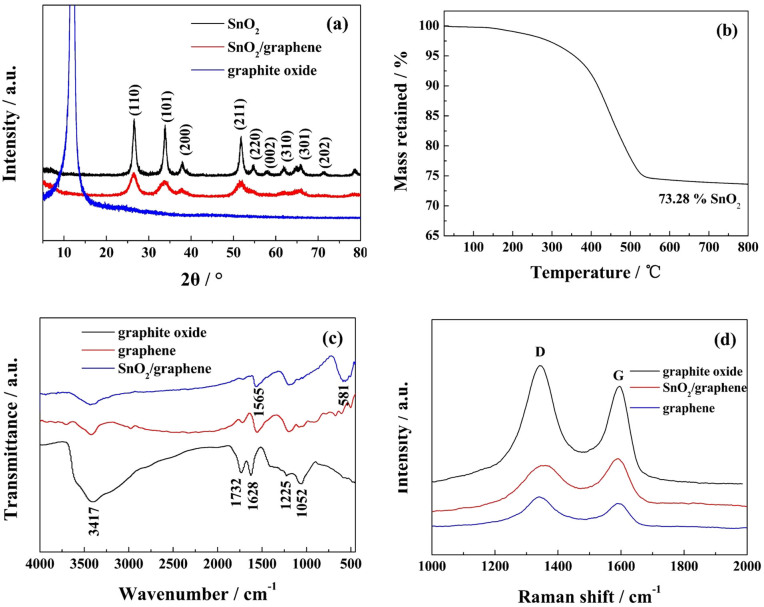
Structures of samples. (a) XRD patterns of SnO_2_, graphite oxide and SnO_2_/graphene, TGA curve of SnO_2_/graphene composite measured in air atmosphere with a heating rate of 20°C min^−1^, (c) FT-IR spectra and (d) Raman spectra of graphite oxide, graphene and SnO_2_/graphene.

The structures of materials were systematically research. The X-ray diffraction (XRD) patterns of the prepared products are illustrated in figure 1 (a). The diffraction peaks of crystalline SnO_2 _nanoparticles are clearly distinguishable. All strong diffraction peaks are consistent with a tetragonal crystalline SnO_2_ phase (JCPDS card no. 41–1445). No diffraction peaks of graphene and graphite oxide are observed in the SnO_2_/graphene composite, indicating that graphite oxide is reduced to graphene and the graphene layer is exfoliated completely^14^,^25^,^31^. This observation helps support the inference that graphene oxide is reduced in situ by bivalent tin ions. For quantifying the content of SnO_2_ in the SnO_2_/graphene composite, thermogravimetric analysis (TGA) was carried out in air. The sample was heated from 25 to 800°C at a rate of 20°C min^−1^. The TGA curve of the SnO_2_/graphene composite is shown in figure 1 (b). From 250 to 550°C,the weight loss increases dramatically with the rise of temperature, attributed to the oxidation and decomposition of graphene. After 700°C,there is no obvious mass loss. From the result, we can approximately calculate the mass content of SnO_2_ in the composite is 73.28%. The Fourier transform infrared (FT-IR) spectra of graphite oxide, graphene and SnO_2_/graphene is presented in figure 1 (c). The peak at 3417 cm^−1^ can be attributed to O-H stretching vibrations of adsorbed water molecules and structural −OH groups. In the graphite oxide spectrum, the peak at 1628 cm^−1^ can be attributed to O-H bending vibrations. Carboxyl and epoxy functional groups can also be detected at approximately 1732, 1225, and 1052 cm^−1^, respectively. Compares to the FT-IR spectrum of graphite oxide, those of graphene and SnO_2_/graphene show that carboxyl group peaks at 1732 cm^−1^ decrease significantly, while carboxyl group peaks at 1225 cm^−1^ and those of epoxy functional groups at 1052 cm^−1^ disappeared. Two strong peaks of SnO_2_/graphene are detected at 581 and 1565 cm^−1^ that corresponds to Sn-O-Sn antisymmetric vibrations and skeletal vibrations of the graphene sheets, respectively^32^. The Raman spectra of graphite oxide, graphene and SnO_2_/graphene are shown in figure 1 (d). The peak at approximately 1581 cm^−1^ (G band) corresponds to the E_2g_ mode of graphite, which is related to the vibration of sp^2^-bonded carbon atoms in a 2-dimensional hexagonal lattice. The peak at approximately 1346 cm^−1^ (D band) is an indication of defects associated with vacancies, grain boundaries and amorphous carbon species^33^. Raman spectra with characteristic G and D bands are sensitive to defects, disorder and carbon grain size, and have been used extensively in the characterization of carbon materials. The intensity ratio (*I*_D_/*I*_G_) of the D band to the G band is related to the extent of disorder degree and average size of the sp^2^ domains^34^. From figure 1 (d), the *I*_D_/*I*_G_ of SnO_2_/graphene is lower than those of graphite oxide and graphene sheets, indicating that bivalent tin can increase the order degree of graphene layers.

**Figure 2:**
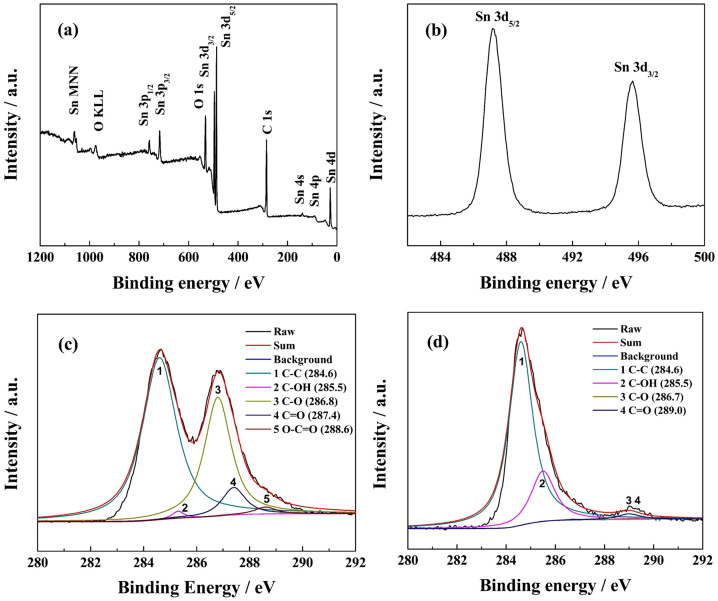
XPS spectra of graphene and SnO_2_/graphene. (a) general XPS spectrum of SnO_2_/graphene, (b) Sn 3d XPS spectrum of SnO_2_/graphene, (c) C 1s XPS spectra of graphite oxide and (d) C 1s XPS spectra of SnO_2_/graphene.

The surface chemistry of the SnO_2_/graphene composite obtained from the X-ray photoelectron spectroscopy (XPS) is presented in figure 2. Figure 2 (a) shows the general XPS spectrum of SnO_2_/graphene, which reveals the presence of carbon, oxygen and tin, and no other elements are detected. The peaks of Sn 3d, 4d, 3p, 4p and 4s from SnO_2_ are observed. The peak of C 1s is attributed mainly to graphene. The Sn 3d spectrum, as shown in figure 2 (b), two peaks at 487.2 and 495.6 eV are attributable to Sn 3d_5/2_ and Sn 3d_3/2_ spin-orbit peaks of SnO_2_, confirming the formation of SnO_2 _nanoparticles on the surface of graphene sheets. Figure 2 (c) and (d) display the spectra of C 1s of graphite oxide and SnO_2_/graphene. The C 1s region of graphite oxide gives five components at around 284.6, 285.5, 286.8, 287.4 and 288.6 eV, which can be generally assigned to the C-C, C-OH, C-O, C = O and O-C = O components, respectively^35^. In contrast, SnO_2_/graphene exhibits dramatically decreased intensities of the C 1s components associated with carbon-oxygen bond, which indicates that most of the oxygenated functional groups on graphene oxide have been removed during the microwave-hydrothermal process. A few residual functional groups are reported to be useful in obtaining stable, highly dispersed SnO_2_ particles^36^.

**Figure 3:**
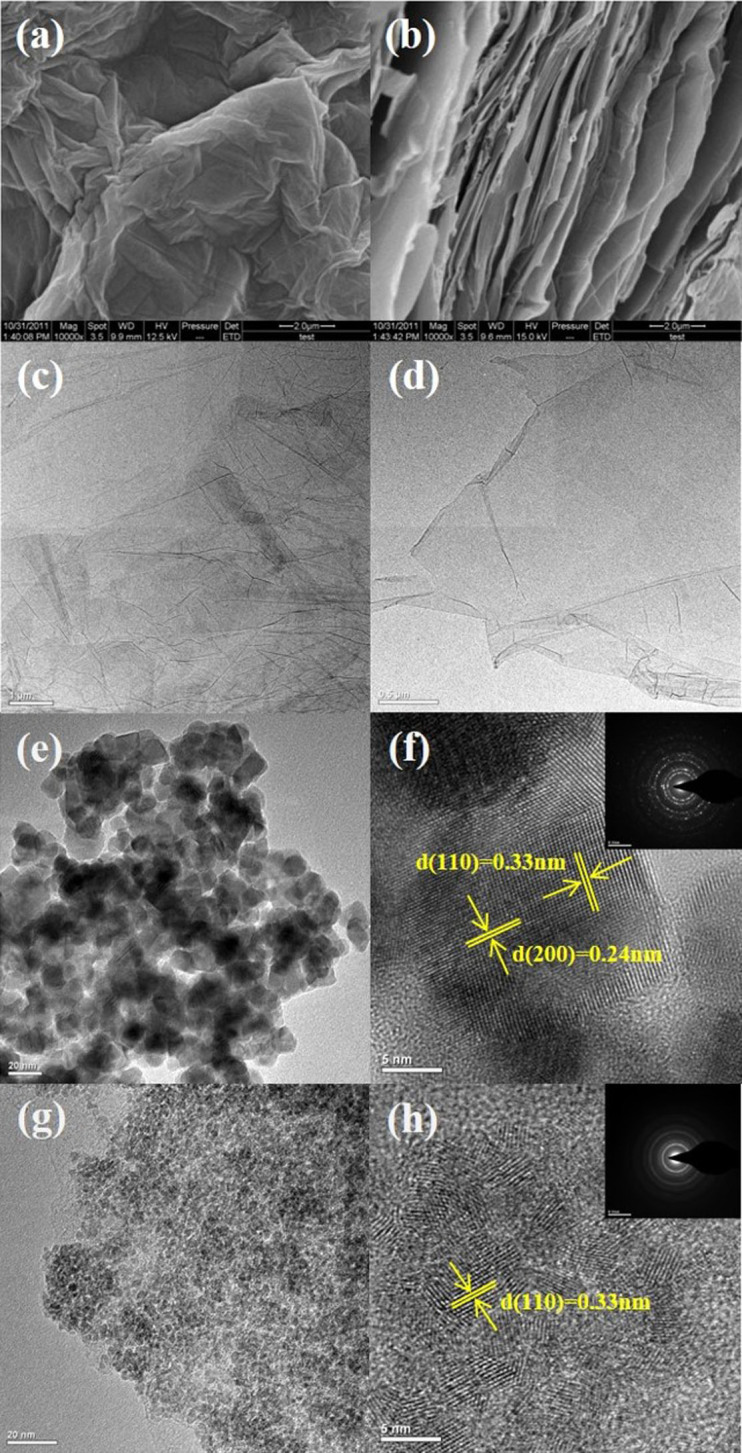
Morphology characterization of graphite oxide, graphene, SnO_2_ nanoparticles and SnO_2_/graphene. (a)(b) SEM images of graphite oxide, (c) (d) TEM images of graphene sheets, (e) TEM images of SnO_2_ nanoparticles, (f) High resolution transmission electron microscopy (HRTEM) images of SnO_2_ nanoparticles, (g) TEM images of SnO_2_/graphene, and (h) HRTEM of SnO_2_/graphene.

The morphologies of graphite oxide, graphene sheets, SnO_2_ and the SnO_2_/graphene composite are observed by scanning electron microscope (SEM), transmission electron microscopy (TEM) and high resolution transmission electron microscopy (HRTEM). Figure 3 (a) and (b) present the SEM images of graphite oxide, showing the layered platelets are composed of curled and wrinkled graphene sheets. It is obvious that the graphene sheets are agglomerated and overlapped. Figure 3 (c) and (b) show TEM images of graphene sheets prepared using the one-step microwave-hydrothermal method. The larger graphene sheet is transparent and resembles a piece of gauze. From figure 3 (e), SnO_2_ nanoparticles prepared using the microwave-hydrothermal method aggregated into large particles, and the average particle diameter of SnO_2 _nanoparticles is 13 nm. These large particles can be pulverized easily owing to an asymmetric volume change during the Li^+^ insertion/extraction process^37^,^38^. However, the TEM image of SnO_2_/graphene in figure 3 (g) shows that the graphene sheets is covered by ultrafine SnO_2_ nanoparticles and the diameter of the SnO_2_ grains is less than 3.5 nm, indicating that the graphene sheets can prevent the SnO_2_ nanoparticles from growing effectively. From the HRTEM images in figure 3 (f) and (h), the interplanar distances of 0.33 and 0.24 nm can be identified as d (110) and d (200) of SnO_2_ nanoparticles, respectively. According to electron diffraction patterns (insets of figure 3 (f) and (h)), the four distinct diffraction rings represent (110), (101), (200), and (210) from the rutile phase of SnO_2_, confirming the highly crystalline nature of SnO_2_ nanoparticles^39^,^40^.

**Figure 4:**
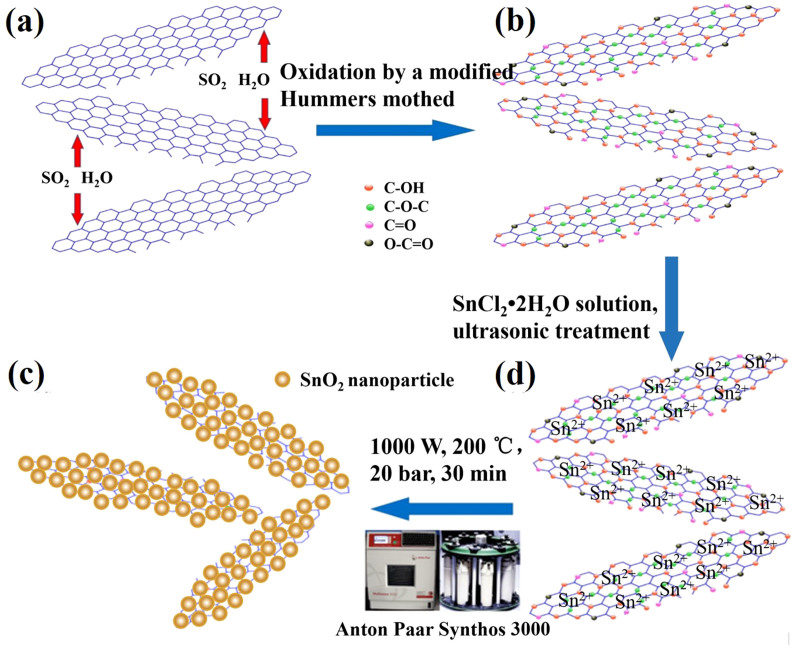
Schematic representation of the fabrication process of SnO_2_/graphene. (a) Expanded graphite, (b) Graphene oxide, (c) Graphene oxide and SnCl_2_·2H_2_O mixed solution and (d) SnO_2_/graphene composite.

According to the microstructural characterization results, a possible formation mechanism for the one-step microwave-hydrothermal reaction without using organic solvent or surfactant is described in the schematic representation of the fabrication process of SnO_2_/graphene (figure 4) and the following equations:


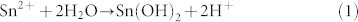

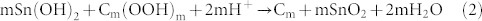
The graphene oxide is prepared by modified Hummers method with the expanded graphite as a material. The larger layer spacing of expanded graphite can improve the efficiency of oxidation, the degree of oxidation and stripping of graphene oxide. There are plenty of oxygen containing functional groups on the surface and edge of graphene sheets. The bivalent tin ion underwent hydrolysis and rapidly transforms to Sn(OH)_2 _with the assistance of microwave heating. Then Sn(OH)_2 _is oxidized to SnO_2 _by oxygen from the oxygenated functional groups of graphene oxide in the Teflon vessels at 200°C. The precursor liquid graphene oxide loses oxygen atoms and is reduced to graphene sheets in situ. In sealed high pressure Teflon vessels of the microwave reaction system, when microwave radiates graphene oxide and SnCl_2_·2H_2_O mixed solution, graphene oxide and water molecules transform their orientation quickly with the microwave frequency. At the same time, Sn^2+^ and hydrated ion of graphene oxide solution transfer back and forth, and strike with adjacent molecules under the function of electric field force. The total energy of the molecules is increased due to molecules rotate and collisions of friction. Therefore the solvent can be brought to a temperature well above its boiling point by the increase of pressure resulting from heating, which is beneficial to the rapid nucleation of nanoparticles. In addition, overheated supercritical water can also play the role of reducing agent and offers a green chemistry alternative to organic solvents during this reaction. The physiochemical properties of overheated supercritical water can be widely changed with changes in high pressure and high temperature, which allow the catalysis of a variety of heterolytic (ionic) bond cleavage reactions in water^41^. Under high temperature and pressure, the SnO_2_ nanoparticles can distribute on graphene sheets uniformly and form nanoporous composite with a large number of void spaces. The large number of void spaces can buffer large volume changes of SnO_2_ nanoparticles during the lithium ion insertion/extraction process^12^,^18^,^42^. The graphene sheets are distributed between the SnO_2 _nanoparticles, preventing the aggregation of these nanoparticles to a certain extent^22^,^23^. Moreover, SnO_2 _nanoparticles on the surface of graphene can also prevent the graphene from stacking into multilayers.

### Electrochemical properties

**Figure 5:**
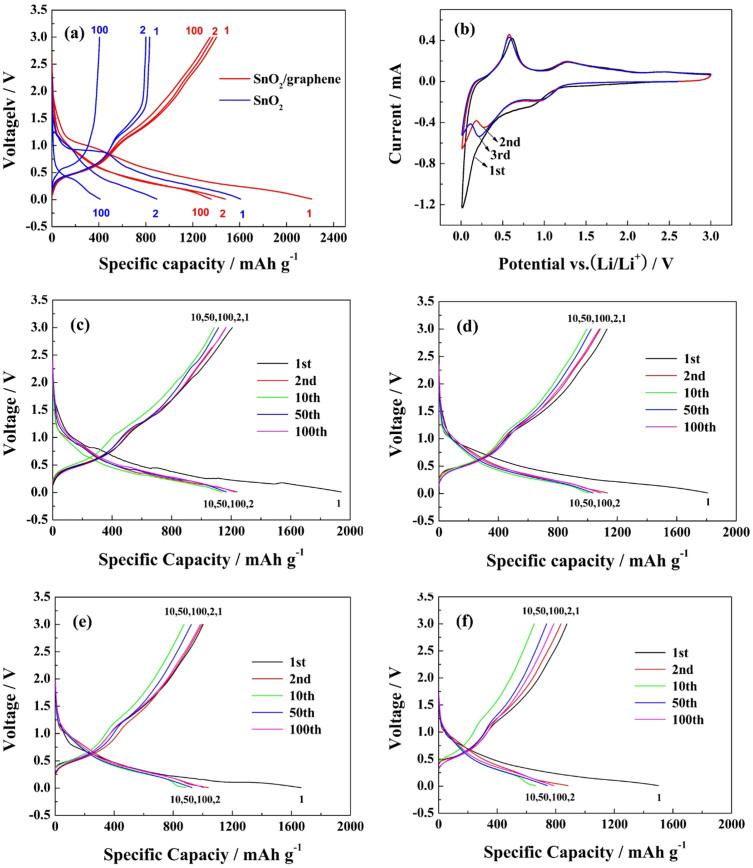
Discharge/charge profiles and CV curves. (a) Discharge/charge profiles of SnO_2_ and SnO_2_/graphene at a current density of 100 mA g^−1^, and (b) CV curves of SnO_2_/graphene at a scanning rate of 0.1 mV s^−1^, (c) Discharge/charge profiles of SnO_2_/graphene at 300 mA g^−1^, (b) at 500 mA g^−1^, (c) at 700 mA g^−1^, and (d) at 1000 mA g^−1^.

In order to study the lithium storage performances and mechanism of electrode material, the LIBs of SnO_2_/graphene composite as an anode material were tested. Figure 5 (a) shows the 1st, 2nd and 100th discharge and charge curves of SnO_2_ and SnO_2_/graphene at a current density of 100 mA g^−1^ in the voltage range of 3.0 ~ 0.0 V vs. Li^+^/Li. In the first cycle, the discharge/charge capacities of SnO_2_/graphene and SnO_2_ are approximately 2213/1402 and 1607/833 mA h g^−1^ with coulomb efficiencies of 66.74 and 51.84%, respectively. The initial small plateau in the potential range of 1.2 to 0.8 V corresponds to a classical conversion reaction between SnO_2_ and Li^+^ and resulted in the formation of Sn and Li_2_O in the first discharge of SnO_2_/graphene, which corresponds to the cathodic peak at approximately 0.85 V. The plateau nearly disappeared during the second cycle, demonstrating that a large amount of Li_2_O is formed during the first cycle. Because of this irreversible reaction, as well as the solid electrolyte interface (SEI) formed on the anodes^43^,^44^, the discharge capacity dropped from 2213 to 1402 mA h g^−1^, and the discharge capacity of SnO_2_ dropped more significantly.

The Cyclic voltammetry (CV) curves of SnO_2_/grapehen is shown in figure 5 (b). In the first cycle, two obvious cathodic peaks appeared around 0.85 and 0.02 V. The peak around 0.85 V is ascribed to the formation of SEI layers on the surface of the active materials, the reduction of SnO_2_ to Sn, and the synchronous formation of Li_2_O (Eq. (3))^11^,^14^. The peak at approximately 0.02 V corresponds to the formation of a series of Li_x_Sn alloys (Eq. (4))^23^. In the first anodic process, there is a small peak near 0.17 V, which can be attributed to Li intercalation into graphite to form LiC_6_ (Eq. (5))^45^. There are also two obvious plateaus at 0.61 and 1.28 V, which can be ascribed to Li dealloying from Li_x_Sn and the partially reversible reaction from Sn to SnO_2_, respectively. The CV measurements clearly elucidated the reversible electrochemical reactions between the lithium ions and the SnO_2_/graphene composite in lithium ion cells. The reactions are described in the following equations:
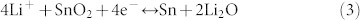

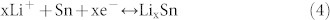

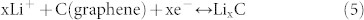
Figure 5 (c) ~ (f) present typical discharge and charge profiles of the SnO_2_/graphene composite at different current densities of 300, 500, 700 and 1000 mA g^−1^, with a voltage range of 3.0 ~ 0.01 V. As shown in figure 5 (c), the initial discharge and charge capacities at a current density of 300 mA g^−1 ^are 1940 and 1224 mA h g^−1^, respectively. When the current density increases to 500 and 700 mA g^−1^, the discharge/charge capacities are 1810/1133 and 1660/1003 mA h g^−1^, respectively, as shown in figure 5 (d) and (e). Even at a current density of 1000 mA g^−1^, the initial discharge and charge capacities are 1502 and 876 mA h g^−1^, as shown in figure 5 (f). The coulomb efficiencies of the first cycle are 63.09, 62.60, 60.42 and 58.32%, and all the coulomb efficiencies increased above 98% after 6 cycles. These values indicates that the SnO_2_/graphene composite can exhibit superior lithium storage properties and that the lithium insertion and extraction reactions are highly reversible.

**Figure 6:**
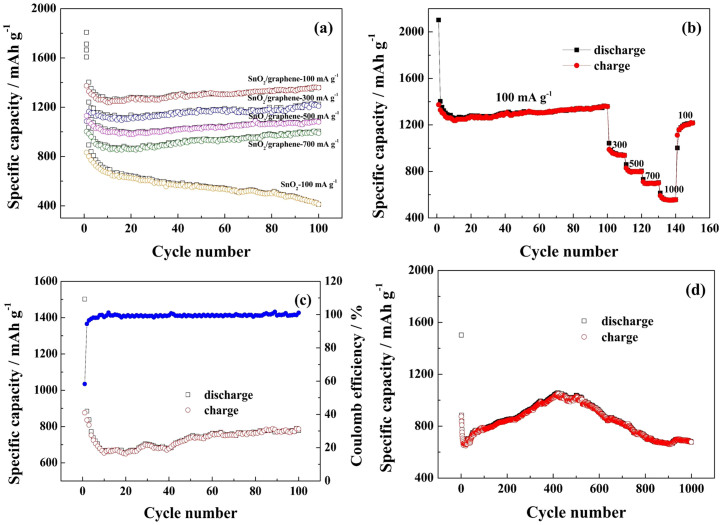
Cycling performances and rate performances. (a) Cycling performance of SnO_2_/graphene between 3.0 and 0.01 V at different current densities of 100, 300, 500, 700 mA g^−1^, (b) Rate performance of SnO_2_/graphene, (c) Cycling performances and coulomb efficiency at 1000 mA g^−1 ^in 100 cycles, and (d) Cycling performances at 1000 mA g^−1 ^in 1000 cycles.

Figure 6 shows the cycling performance and rate performance of the SnO_2_/graphene. The discharge specific capacity at a current of 100 mA g^−1^ is 1359 mA h g^−1^ after 100 cycles. We can see from figure 6 (a) that the discharge specific capacities are still as high as 1228, 1090, 1005 and 790 mA h g^−1^ at current densities of 300, 500, 700 and 1000 mA g^−1^ after 100 cycles, respectively. However, the discharge specific capacity of bare SnO_2_ significantly decreases. In addition, the capacities of the composite are higher than the theoretical specific capacities of SnO_2 _(782 mA h g^−1^) and graphene (744 mA h g^−1^), and all the discharge specific capacities at different current densities begin to increase after the 19th cycle. In order to examine the rate performance of SnO_2_/graphene electrode towards different current densities, the SnO_2_/graphene electrode was cycled at different current densities (300, 500, 700, 1000 mA g^−1^) after 100 cycles at 100 mA g^−1^, and reversed back to low current density of 100 mA g^−1^. We can see from figure 6 (b) that the capacity decreases with increasing current densities. When the current density was reversed back to 100 mA g^−1^, the capacity almost recover to the original value. This indicates that the SnO_2_/graphene is quite suitable for large current charge and discharge. The cycling performance and coulomb efficiency of SnO_2_/graphene at 1000 mA g^−1^ are shown in figure 6 (c) and (d). The discharge specific capacity at the current density of 100 mA g^−1^ is 778 mA h g^−1^ after 100 cycles. The first coulomb efficiency is 58.3%, and then ~ 99.5% in the following cycles. It should be noted that the capacity increases with increasing cycles of discharge and charge until 1057 mA h g^−1 ^at 420th cycling, and then decreases gradually. The discharge specific capacity is stability and remains 677 mA h g^−1 ^after 1000 cycles.

**Figure 7:**
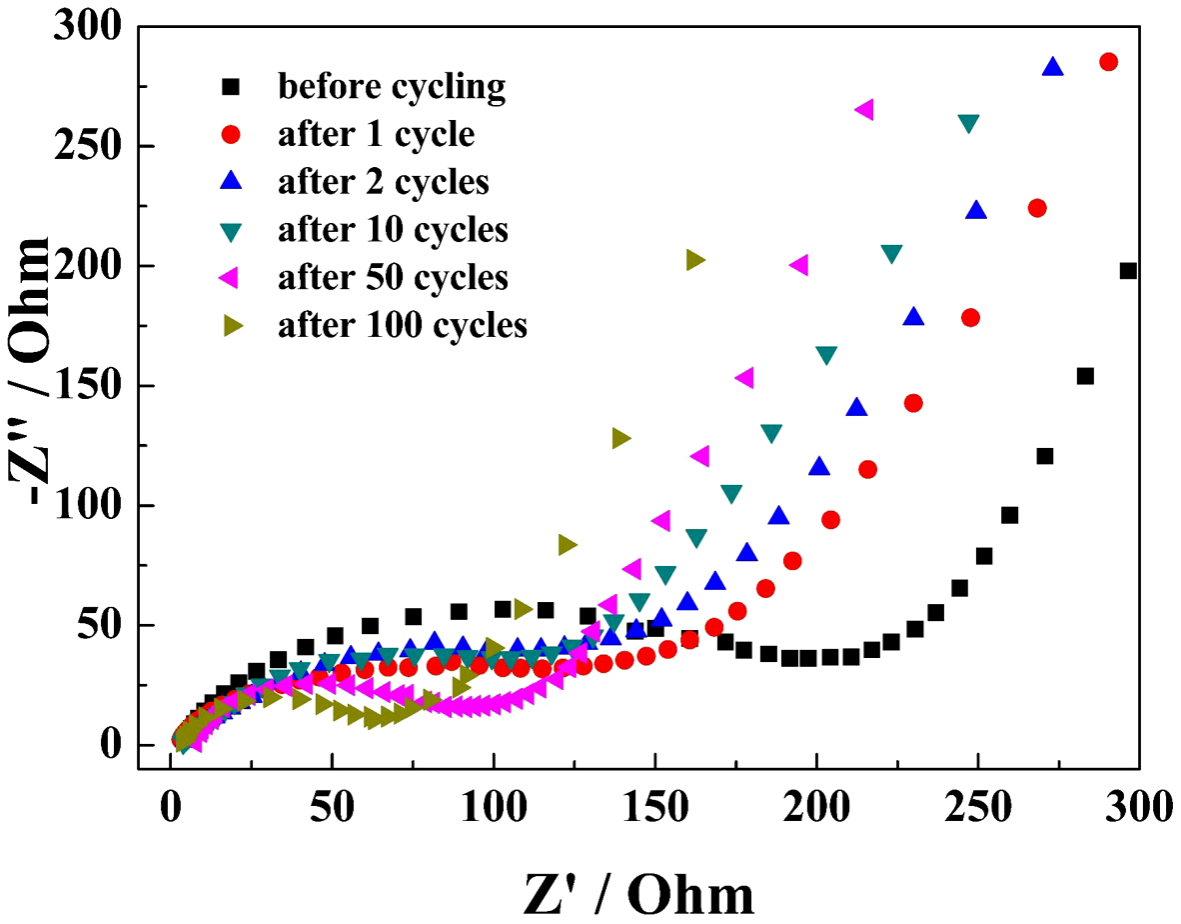
EIS of SnO_2_/graphene before and after 1, 2, 10, 50, 100 charge/discharge cycles.

Figure 7 shows electrochemical impedance spectroscopy (EIS) for SnO_2_/graphene before and after 1, 2, 10, 50, 100 charge/discharge cycles. The high-frequency semicircle is attributed to the constant phase element of the SEI film and contact resistance. The semicircle in the medium-frequency region is assigned to charge-transfer impedance and the constant phase element of the electrode/electrolyte interface. The inclined line is associated with Warburg impedance corresponding to the lithium-diffusion process. The diameters of the semicircles in both high and medium frequency areas become noticeably smaller evidently after one cycle, indicating that the first several cycles are the electrode activation process and the decrease in the impedance value of SnO_2_/graphene electrode. The resistances of SnO_2_/graphene decrease gradually due to the size of Sn nanoparticles decrease gradually after 10, 50 and 100 cycles. The decreased resistance can enhance ionic conductivity in the composite, which is beneficial for Li^+^ insertion/extraction into the anodes^23^,^30^,^46^,^47^.

## Discussion

The reversible capacities only decrease in the first 19 cycles and then exhibit a slight increase with additional discharge/charge. The decay of reversible capacities of the SnO_2_/graphene during the first 19 cycles can be attributed to the pulverization of original SnO_2_ and in situ formed Sn nanoparticles during Li insertion and extraction process, which lead to loss of electrical connectivity between neighboring particles^12^. In the initial cycles, the pulverized particles did not contact well with each other, as a result, the reversible specific capacity decreases. However, the Sn nanoparticles began shrink and pulverize into small particles when Li^+^ was extracted. With Li insertion and extraction, the formed Sn nanoparticles became smaller and smaller due to electrochemical milling effects and strong attached to the graphene sheets. Therefore, the conversion reaction of SnO_2_ in the SnO_2_/graphene composite, SnO_2_ + 4Li^+^ + 4e^−^ → 2Li_2_O + Sn, would be reversible to a certain extent due to the very small Sn nanoparticles. Ahn *et al.* claimed that the decrease of reagent particle size could reduce the activation energy for solid-state double decomposition reaction, thus boosting the conversation reaction and contributing to the reversible capacity^48^.

**Figure 8:**
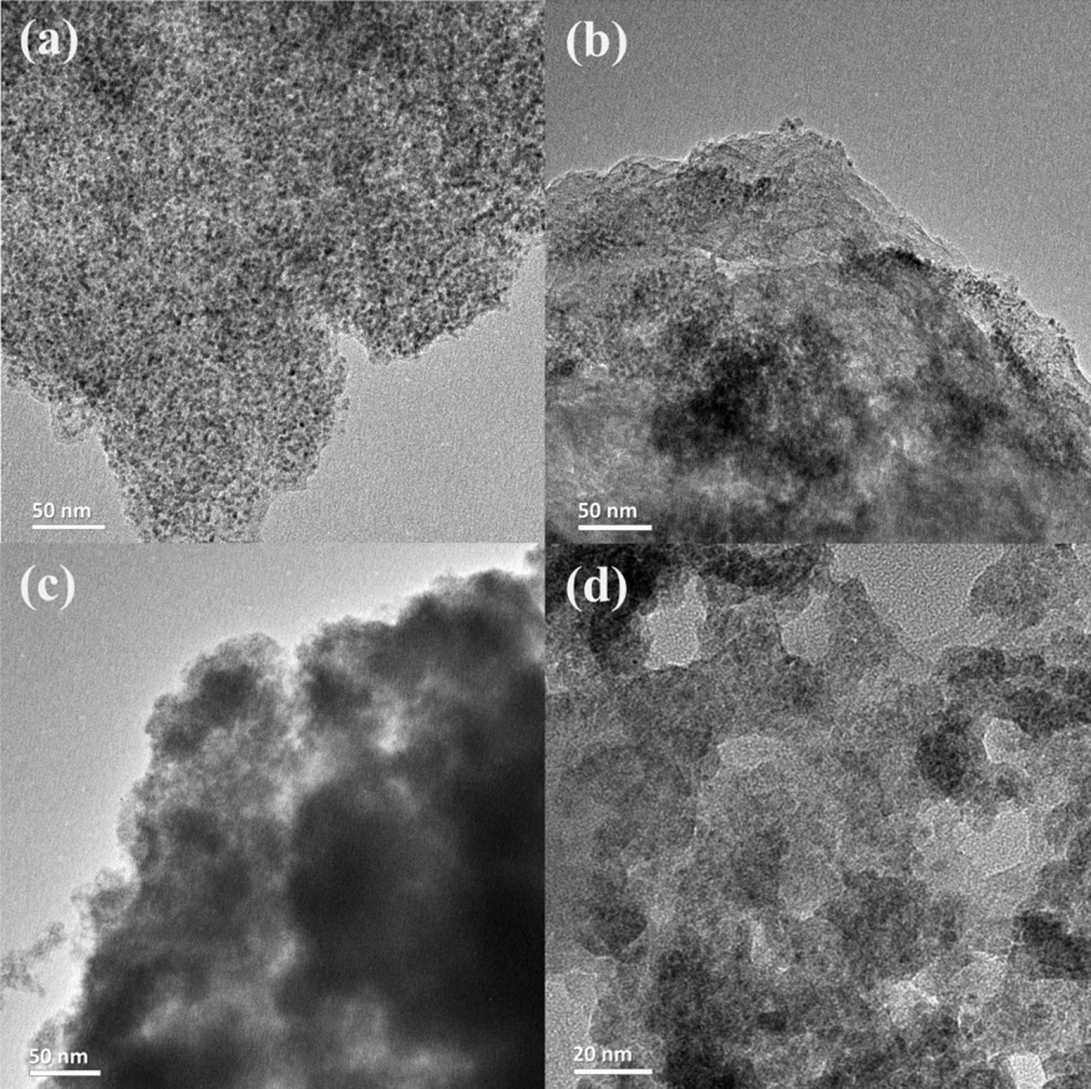
TEM images of SnO_2_/graphene composite after different discharge/charge cycles at a current density of 1000 mA g^−1^. (a) After 20 charge/discharge cycles, (b) After 100 charge/discharge cycles, (c) After 400 charge/discharge cycles, and (d) After 1000 charge/discharge cycles.

The cycling performances are almost superior to most SnO_2_/graphene composites, especially at high current densities. The excellent reversible capacities are also attributed to the critical size of SnO_2_ nanoparticles^49^. It is reported that the particle size is one of the key factors for the stable cycling performance of SnO_2_, where smaller particle size can help to prevent gradual aggregation of Sn into large clusters^50^. Kim et al. proved that particles with larger sizes are more vulnerable to aggregating into tetragonal Sn clusters, whereas ~ 3 nm sized SnO_2_ shows no aggregation upon cycling with cubic Sn formation^51^. In order to explore the root of the excellent cycling stability and high capacity of the SnO_2_/graphene composite, the morphologies of electrode materials after cycling performance testing were studied. Figure 8 shows the TEM images of SnO_2_/graphene composite after 15, 100, 400 and 1000 cycles of discharge/charge test at 1000 mA g^−1^. As can be seen from figure 8 (a), SnO_2_ nanoparticles are uniformly distributed on the graphene sheets, indicating that SnO_2_/graphene composite can maintain its structure after 15 cycles. After 100 cycles, the average size of nanoparticles become small, but the boundaries of SnO_2_ nanoparticles are still clear (figure 8 (b)). After 400 cycles, the nanoparticles no longer show clear boundaries because of repeated lithium alloying and dealloying (figure 9 (c)). But the capacity of SnO_2_/graphene composite in the 400^th^ cycle shows no fade, which is similar with reported in literatures^52^,^53^,^54^. All these phenomena reveal that the interaction between SnO_2_ nanoparticles and graphene sheets is very strong before 400 cycles of discharge/charge test at 1000 mA g^−1^, and reversible reaction of SnO_2_ in the SnO_2_/graphene composite is more and more thoroughly due to the size of nanoparticles become smaller and smaller. Therefore, the specific capacity of SnO_2_/graphene increases during the first 400 cycles in cycling performances measurement at 1000 mA g^−1^. Figure 8 (d) shows the TEM images of SnO_2_/graphene composite after 1000 cycles. The size of SnO_2_ nanoparticles become large and the surface of the nanosheets becomes textured and big holes, indicating the SnO_2_ nanoparticles begin peeling off after long cycles due to the large volume changes. As a result, the specific capacity gradually decreases after 420^th^ cycling. But it is worth noting that the reversible capacity of SnO_2_/graphene composite is stability at last. Chen *et al*. reported that the porous nanostructure have more edges, which were able to provide more activated sites for Li storage and also facilitated the penetration of the electrolytes^49^. Fan *et al.* claimed that the theoretical reversible capacity for porous graphene with more edges for Li storage could reach as high as 1965 mA h g^−1^^55^. Therefore, such excellent cycling performances may be related to the porous network structure of the SnO_2_/graphene composite. The porous structure can facilitate liquid electrolyte diffusion into the electrode material. Meanwhile, SnO_2_ nanoparticles in the as-prepared SnO_2_/graphene composite can reduce the path length for Li^+^ transport and the porous graphene sheets can increase theoretical lithium storage capacity. Therefore, although the structure of SnO_2_/graphene composite have changed after prolonged cycling process, the reversible capacity of SnO_2_/graphene composite is stability and remains 677 mA h g^−1^ after 1000 cycles at the large current density of 1000 mA g^−1^.

In summary, a SnO_2_/graphene composite has been synthesized successfully by a one-step microwave-hydrothermal method. This method features quick heating, easily controlled pressure and temperature, high yield rate, and good homogeneity. In the reaction process, liquid graphite oxide as a precursor material is reduced more fully in situ, and bivalent tin ion is oxidized under microwave and high pressure with the assistance of bivalent tin and without auxiliary reagents. SnO_2_ nanoparticles of ~ 3 nm distributes on the graphene uniformly and formed nanoporous composites with large numbers of void spaces. The uniform composite shows high specific capacity and excellent cycling stability performance for LIBs at high current densities. The superior Li+ storage performances of SnO_2_/graphene composite as anode materials for LIBs are attributed to the following benefits. (1) The critical size of SnO_2_ nanoparticles ensures the isolation of nanoparticles with cubic Sn formation and is beneficial for the suppression of their aggregation during electrochemical discharge/charge process, resulting in improved cycling stability. (2) The uniform SnO_2_ nanoparticles in the as-prepared SnO_2_/graphene composite can reduce the path length for Li^+^ transport and supply large specific surface, which is suitable for large current density charge and discharge. (3) The strong contact between SnO_2_ nanoparticles and graphene sheets can guarantee the stability of the SnO_2_/graphene composite, which can suppress peel off of SnO_2_ nanoparticles and increase the electronic conductivity. (4) The porous network structure of the SnO_2_/graphene composite can increase theoretical lithium storage capacity and abundant active sites for full utilization of active materials, also can facilitate liquid electrolyte diffusion into the electrode material, which is beneficial for achieve high reversible capacity. The results of electrochemical measurement show that the SnO_2_/graphene composite synthesized by the one-step microwave-hydrothermal method is a promising anode material for LIBs.

## Methods

### Preparation of graphite oxide and graphene oxide

Graphene oxide solution was synthesized by a modification to Hummer's method. Briefly, 2 g expanded graphite was added to a 500 mL beaker, followed by 150 mL concentrated sulfuric acid; the beaker was then placed in an ice-water bath and stirred for 10 min. Potassium permanganate (8 g) was added slowly and stirred for 30 min, then the beaker was placed in a water bath at 35°C and stirred with a mechanical stirrer for 24 h. Deionized water (100 mL) was added to the beaker at 98°C and stirred for 10 min. Then 40 mL H_2_O_2_ (30%) was added to the mixture and continuously stirred for 1 h. The mixture was filtered and washed with 10% HCl and 1% H_2_O_2_ solutions until the pH was 5. Graphite oxide of 1 mg mL^−1^ as a precursor material was exfoliated by high power ultrasonication for 30 min.

### Preparation of SnO_2_/graphene composite

The SnO_2_/graphene composite was prepared by a one-step microwave-hydrothermal method. In a typical reaction, 35 mL 0.04 mol L^−1^ SnCl_2_·2H_2_O solution (0.3159 g SnCl_2_·2H_2_O and 35 mL deionized water) was added to 120 mL 1 mg mL^−1^ graphene oxide aqueous solution, followed by ultrasonic treatment for 10 min. The brown supernatant solution was transferred to high pressure Teflon vessels of the microwave reaction system (Anton Paar Synthos 3000). The system power, temperature, pressure and reaction time were 1000 W, 200°C, 20 bar and 30 min, respectively. The black as-synthesized product was cleaned several times by centrifugation with ethanol and deionized water.

### Preparation of SnO_2_ nanoparticles and graphene sheets

As comparison, graphene sheets were prepared with a one-step microwave-hydrothermal method under the same parameters without the addition of SnCl_2_·2H_2_O. Pure SnO_2_ was prepared by a microwave-hydrothermal method, then 15 mL polyethylene glycol (MW = 400) was added to 30 mL SnCl_4_·5H_2_O solution with concentration of 0.04 mol L^−1^; the pH was adjusted by ammonia. The autoclave was sealed and placed in a microwave reaction system at 200°C for 60 min. The hoary solid product was annealed under 500°C for 2 h.

### Sample characterization

The structure and morphology of the as-prepared materials were characterized by SEM (QUANTA 200F), TEM (FEI TECNAI G2 F20), TGA (HITACHI, STA7300), and XRD (Bruker D8 Advance with Cu Kα radiation) operated at 40 kV and 40 mA. Raman (Renishaw RM-1000) were recorded in a plus laser Raman spectrometer with an excitation laser beam wavelength of 514.5 nm. FT-IR analysis was carried out using pressed KBr disks in the range 4000–400 cm^−1^ with a PerkinElmer spectrometer. XPS (VG Scientific ESCALAB 2201XL) was carried out using Al Kα X-ray radiation and fixed analyzer transmission mode.

### Electrochemical measurements

Electrochemical measurements were carried out using CR2025 coin-type cells. The working electrode was prepared by coating slurries consisting of the active material, polyvinylidene fluoride and acetylene black with a weight ratio of 80:10:10 in N-methyl-pyrrolidone solvent. The slurries were uniformly pasted on a copper foil thin film and dried at 120°C in a vacuum oven for 12 h. The thin film on copper foil was cut into round disks with diameter of 12 mm and pressed under a pressure of approximately 200 kg cm^−2^. The films were then dried at 120°C in a vacuum oven for 3 h and used as anodes for the coin cells. The weight of working electrode materials is ~ 1.15 mg, and the weight of SnO_2_/graphene composite in a coin cell for testing is 0.9124 mg. The coin cells were assembled inside a glove box filled with pure argon, using lithium metal as the counter/reference electrode and Celgard2325 as the separator. The electrolyte was 1 M LiPF_6_ dissolved in a mixture of dimethyl carbonate, diethyl carbonate and ethylene carbonate (1:1:1 by weight). Galvanostatic charge-discharge curves of the cells were recorded by a Battery Testing System (Neware Electronic Co., China) at various current densities from 100 to 1000 mA h g^−1^ with the voltage between 3.0 and 0.01 V *versus* Li^+^/Li at room temperature. CV curves were measured from 0.01 to 3.0 V at a scanning rate of 0.1 mV s^−1^, and EIS data were obtained by applying an AC voltage of 5 mV in the frequency range of 0.01–100 kHz using an electrochemistry working station (AUTOLAB PGSTAT302).

## Author Contributions

M.Z.A. originated the work and designed the experiments. L.L.L. performed the experiments, analyzed the data and wrote the manuscript. P.X.Y. performed XPS and XRD measurement and data analysis. J.Q.Z. carried out cyclic voltammetry and electrochemical impedance spectroscopy measurement and data analysis. All authors reviewed the manuscript.
